# Increased HBV Coinfection and Decreased IFN-γ-Producing HBV-Specific CD8+ T Cell Numbers During HIV Disease Progression

**DOI:** 10.3389/fimmu.2022.861804

**Published:** 2022-03-30

**Authors:** Zhiqiang Zhu, Yuanyuan Qin, Qi Liang, Wei Xia, Tong Zhang, Wen Wang, Mengmeng Zhang, Taiyi Jiang, Hao Wu, Ye Tian

**Affiliations:** ^1^ Department of Urology, Beijing Youan Hospital, Capital Medical University, Beijing, China; ^2^ Beijing Key Laboratory for HIV/AIDS Research, Capital Medical University, Beijing, China; ^3^ Department of Clinical Laboratory, Affiliated Hospital of North Sichuan Medical College, Nanchong, China; ^4^ Translational Medicine Research Center, North Sichuan Medical College, Nanchong, China; ^5^ Center for Infectious Diseases, Beijing Youan Hospital, Capital Medical University, Beijing, China; ^6^ Department of Urology, Beijing Friendship Hospital, Capital Medical University, Beijing, China

**Keywords:** HIV, HBV, disease progression, co-infection rate, IFN-γ

## Abstract

**Objective:**

To investigate the characteristics and mechanism of the dynamics of HBV infection with the progression of HIV disease and to explore the different responses of T lymphocytes to HBV in HIV patients in different stages of disease.

**Methods:**

We compared the rates and characteristics of HBV coinfection between 372 early HIV-infected and 306 chronically HIV-infected men who have sex with men (MSM) in the Beijing Youan Hospital from October 2006 to November 2014. We further analysed IFN-γ-producing HBV-specific CD8+ T cells in 15 early HIV-infected individuals and 20 chronic HIV-infected individuals with HBV coinfection.

**Results:**

Twenty-three HBsAg-positive cases were detected among the 372 early HIV-infected patients of this cohort, and the coinfection rate was 6.18%, while 35 HBsAg-positive cases were detected among the 306 chronically HIV-infected patients, with a coinfection rate of 11.44%. The coinfection rate of the chronically HIV-infected patients was significantly higher than that of the early-infected patients (*p*=0.0005). The median CD4+ T cell count in the early HIV infection patients was 445 cells/μL (196-1,030 cells/μL), which was higher than that in the chronic HIV infection patients [358 cells/μL (17-783 cells/μL)] (*p*<0.001). The proportion of IFN-γ-producing CD8+ T cells in early HIV-infected patients was significantly higher than that in chronically HIV-infected patients.

**Conclusion:**

The coinfection rate of HBV in HIV patients increases with HIV disease progression, which might be related to the decreased IFN-γ-producing HBV-specific CD8+ T cell numbers. The closely monitored HBV serum markers from the early stage of HIV infection are warranted.

## 1 Introduction

HIV and HBV coinfections are clinically common because they can both be transmitted sexually. HBV can cause early infection with or without clinical symptoms in healthy adults, but the human immune system can fight and clear the HBV in the very early stage to prevent progression to the chronic phase (1). Chronic HBV infection is common in infants and children due to their immature immune systems, which cannot rapidly eliminate the HBV and allow the infection to develop to the chronic stage (2). At present, there are differing perspectives on the impact of HIV and HBV coinfection on the progression of HIV disease (2, 3). Several studies have found that HBV infection has no impact on HIV progression in the clinic, while others have found that it promotes HIV progression (4–6). Generally, HIV combined with HBV infection has a great impact on disease progression in patients (7); for example, the incidence of cirrhosis and hepatocellular carcinoma increases among patients with HIV-HBV coinfection compared with HIV infection only (7). Therefore, it is of great significance to understand the characteristics of HBV coinfection in HIV patients and the ways to manage it as early as possible.

Many studies are focusing on HIV patients with HBV infection, but the conclusions have been inconsistent (1, 3, 7–10). Some studies have shown that HIV infection has a positive role in the elimination of the HBV (11), while others have discovered increased HBV coinfection among HIV patients. These controversial conclusions might be due to the different HIV-infected patients chosen by each study. HIV attacks the human immune system and causes dramatic changes to it as disease develops, and different CD4+ T cell levels might lead to different outcomes of HBV infection in HIV patients.

Since there are no overt clinical symptoms of patients with early HIV infection, and few patients can be recruited for detailed observation, most studies have focused on chronic HIV patients. Our previous research showed that the rate of HBV coinfection in early HIV-infected patients is significantly lower than that in chronically HIV-infected patients. Based on this, we inferred that, with the development of disease, the ability to respond to and clear the HBV dramatically decreases. IFN-γ-producing CD8+ T cells play an important role in HBV viral clearance (12). Although many scientists and physicians have speculated that HBV clearance is higher in the early phase of HIV than in chronic phase patients, no research has compared the T cell response to HBV between early and chronic HIV patients. Therefore, we compared and analysed the T cell response to HBV of HIV-infected patients in different phases in this study.

To study the related factors of HIV patients coinfected with HBV at different disease stages in the MSM group, the MSM follow-up cohort from Beijing Youan Hospital affiliated with Capital University was used in this study (9). We describe the clinical characteristics of HBV infection in early HIV-infected patients and chronic HIV-infected patients in this study, hoping to provide evidence for the prevention of HBV infection in people living with HIV.

## 2 Materials And Methods

### 2.1 Study Population

The early HIV infection cohort in this study was from an open and prospective cohort of MSM with high-risk behaviours established in the Beijing Youan Hospital, Capital Medical University (9). The cohort recruited MSM who were HIV-negative at the age of 18 years or older and were followed up every 2 months for HIV antibody and HIV RNA testing until a positive HIV antibody conversion occurred from October 2006 to November 2014. The chronic HIV infection cohort was retrospectively collected from consecutive MSM who were diagnosed with chronic HIV infection in the Beijing Youan Hospital, Capital Medical University from October 2006 to November 2014.

The inclusion criteria were as follows: 1) enrolment in either the early HIV infection cohort or the chronic HIV infection cohort; 2) age >18 years; and 3) no history of antiretroviral therapy (ART). The exclusion criteria were as follows: 1) other hepatitis virus (i.e., HAV, HCV, HDV, and HEV) infected patients and 2) patients with opportunistic infections or AIDS-related tumours.

This study was approved by the Ethics Committee of Beijing Youan Hospital. All patients signed an informed consent form before participating in this study.

### 2.2 Methods

#### 2.2.1 Definition

**Early HIV infection:** Patients who were HIV antibody negative but HIV RNA-positive, those with suspicious HIV antibody WB bands who were HIV-RNA positive, or those who showed positive HIV antibody conversion within 6 months during the cohort follow-up.

Chronic HIV infection: Patients who showed positive HIV antibody conversion over 6 months.

#### 2.2.2 Study Design

The HBV serum markers, HIV RNA and CD4+ T cell number of all patients in the two phases of HIV infection cohorts were tested at the time of HIV diagnosis and confirmed afterwards. HBV serum markers of patients in the acute HIV infection cohort were collected for testing at weeks 1, 2, 4, 8, and 12 and then every 3 months thereafter.

#### 2.2.3 Detection of Hepatitis B Virus (HBV) Infection

HBV-specific antigens and antibodies in patient plasma were measured by Elecsys^®^ HBsAg Immunoassay kits (Roche Diagnostics GmbH, Mannheim, Germany), immunoassay analyser cobas e411 kits (Roche Diagnostics GmbH, Mannheim, Germany) and specific ELISA test kits (PRECHEK Bio, Anaheim, USA) according to the manufacturer’s instructions in the clinical laboratory at Youan Hospital. HBV infection is defined as the presence of HBsAg +/- and detectable HBV DNA (13).

#### 2.2.4 Markers of HIV Disease Progression

Absolute blood CD4+ T cell counts were measured by flow cytometry (BD FACSCanto flow cytometer, BD Bioscience, San Jose, CA, USA). HIV RNA was tested by the Amplicor HIV monitor ultrasensitive method with a detection limit of 40 copies/mL of plasma.

#### 2.2.5 Liver Function Tests

Alanine aminotransferase (ALT) and aspartate aminotransferase (AST) levels in patient plasma were tested by a UV-LDH method test kit (Fortress Diagnostics Limited, United Kingdom).

#### 2.2.6 *In Vitro* Stimulation

The frozen peripheral blood mononuclear cells (PBMCs) collected from healthy individuals, early HIV-infected patients who were HBsAg-positive and chronic HIV-infected patients who were HBsAg-positive were cultured (1×10^6^ cells/mL) in 10 mL foetal bovine serum (Gibco Australia Origin, USA) and stimulated with an HBsAg overlapping peptide pool (donated by Prof. Tao Dong, Oxford) for 8 hours. The transport inhibitor Brefeldin A (3 μg/mL, eBioscience) was added into each stimulus condition. The cells were stained with surface antibodies (CD3-PerCP and CD8-FITC, BD Bioscience, San Jose, CA, USA). The surface-stained cells were washed, fixed, and permeabilized using the Permeabilization/Fixation Kit (eBioscience, Waltham, MA, USA) before intracellular cytokine staining with IFNγ-PE, (BD Bioscience, San Jose, CA, USA). The stained cells were fixed with 1% formaldehyde for analysis by a FACS Canto flow cytometer (BD Bioscience, San Jose, CA, USA) within 24 hours.

#### 2.2.7 Statistical Analysis

Data analyses were performed with Statistical Product and Service Solution 16.0 (SPSS for Windows, Version 16.0. Chicago, SPSS Inc.). Data that fit a normal distribution were expressed as the means ± SD, while those that fit a nonnormal distribution were represented by the medians (lower quartile-upper quartile). The comparison of the related data between different groups was analysed by T test or Chi-square test depending on the data type. *P*<0.05 was considered to be statistically significant for all analyses.

## 3 Results

**Figure 1:**
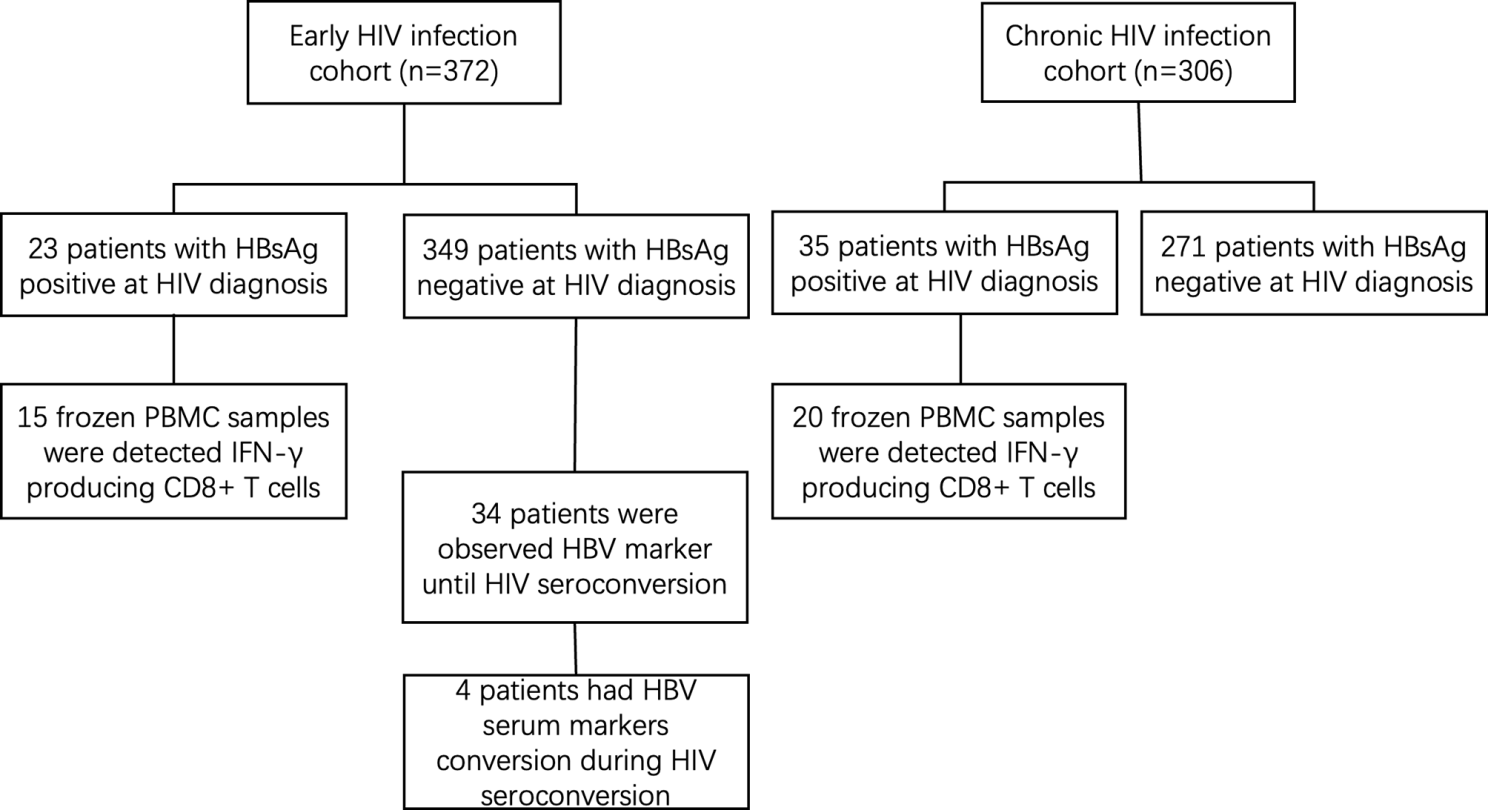
Flow chart of the study procedure.

**Figure 2:**
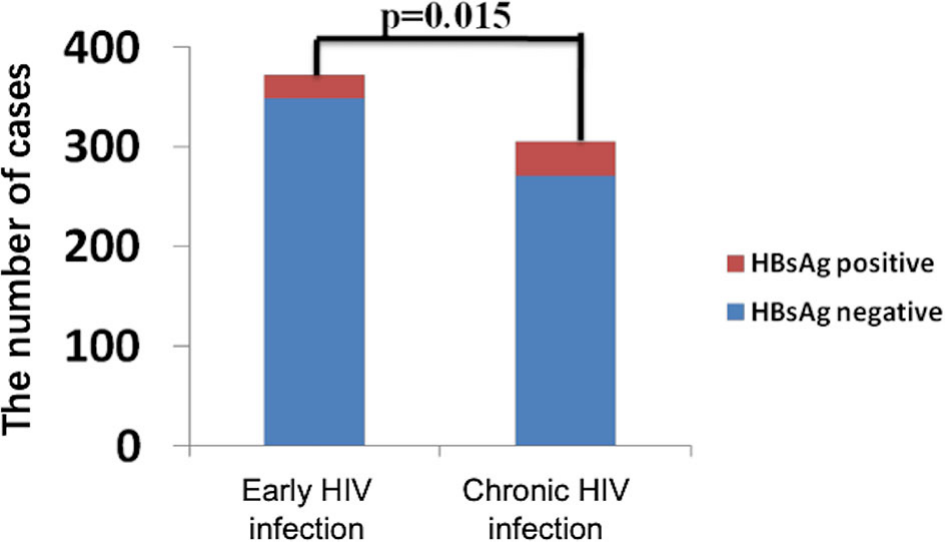
The rate of HBsAg positivity in the early HIV infection and chronic HIV infection groups.

The general information of 372 early and 306 chronically HIV-infected patients was collected from outpatient clinic-based MSM cohorts established in Beijing Youan Hospital, Capital Medical University from October 2006 to November 2014 (**Figure 1**). Twenty-three patients (6.18%) were HBsAg-positive in the 372-patient early HIV infection group, and 35 patients (11.44%) were HBsAg-positive in the 306-patient chronic HIV infection group. The rate of HBV coinfection in the chronic HIV infection group was significantly higher than that in the early HIV infection group (*p*=0.015, **Figure 2**). The CD4+ T cell counts decreased from an average of 445 in the early HIV infection group to 358 in the chronic HIV infection group (*p*<0.001) (**Table 1**). The distributions of age and HIV viral load were not significantly different between the two groups. Additionally, the distributions of HBV coinfection and CD4+ T cell counts were also significantly different between the two groups.

**Table 1:** Characteristics of patients in the early and chronic HIV infection cohorts.

	Early HIV infection(n=372)	Chronic HIV infection(n=306)
Age, years, (median, IQR)	32 (22-59)	33 (19-59)
≤20	17 (4.6%)	10 (3.3%)
21-35	224 (60.2%)	169 (55.2%)
36-50	118 (31.7%)	111 (36.3%)
≥51	13 (3.5%)	16 (5.2%)
HBV coinfection, n(%)		
Yes	23 (6.2%)	35 (11.4%)
No	349 (93.8%)	271 (88.6%)
CD4+T counts, cell/ul, (median, IQR)	445 (196-1030)	358(17-783)
≤200	56 (15.1%)	61 (19.9%)
201-349	132 (35.4%)	132 (43.1%)
≥350	184 (49.5%)	113 (36.9%)
HIV RNA load, copies/ml, (median, IQR)	530, 956 (554-7,280,000)	46,363 (<40-630, 000)

Among the other 349 early HIV-infected patients whose HBsAg was negative at diagnosis, 34 were observed to have HBV serum markers until developing to the chronic phase of HIV infection. During the follow-up period, 4 patients showed HBV-specific antigen and/or specific antibody changes (see **Table 2**). The CD4+ T cell counts decreased and the viral load increased in 4 patients from the early phase to the chronic phase of HIV infection. The number of hepatitis B virus-specific antibodies decreased from the early phase to the chronic phase of HIV infection in the four patients, and two patients’ HBsAg became positive. The ALT and AST levels were similar, and HIV developed from the early to the chronic stage in the four patients.

**Table 2:** Characteristics of patients who had HBV-specific antigens and/or specific antibody changes from early to chronic phases.

Patient	Follow-up	HBsAg	HBsAb	HBeAg	HBeAb	HBcAb	ALT (IU/L)	AST (IU/L)	CD4 (cells/ul)	HIV RNA (copies/ml)
1	Early	_	+	_	_	+	25.0	23.5	430	41,600
	Chronic	_	+	_	_	_	27.3	27.8	251	121,000
2	Early	_	+	_	+	_	27.5	25.4	500	53,800
	Chronic	_	+	_	_	_	20.5	16.6	190	88,200
3	Early	_	+	_	_	_	22.1	24.1	389	222,000
	Chronic	+	_	_	_	_	43.2	36.2	157	415,000
4	Early	_	_	_	_	+	13.0	19.2	420	295,000
	Chronic	+	_	_	_	_	21.6	24.5	170	410,000

**Figure 3:**
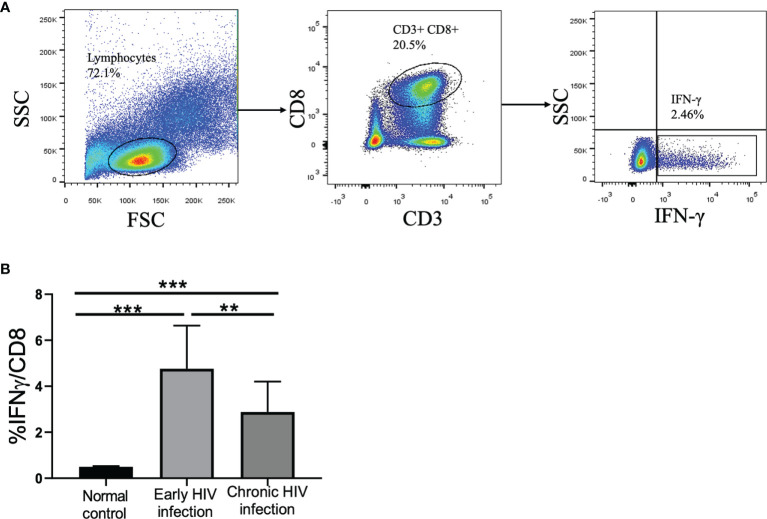
Frequency of IFN-γ-producing CD8+ T cells in normal controls, early HIV infection, and chronic HIV infection groups. **(A)** The gating strategy for flow cytometric analysis of IFN-γ-producing CD8+ T cells. **(B)** Comparison of the proportion of IFN-γ-producing CD8+ T cells. The significance of differences was assessed by calculating P values in Mann–Whitney tests. **p < 0.01, ***p < 0.001.

Subsequently, the proportion of IFN-γ-producing CD8+ T cells in PBMCs was detected in 15 early HIV-infected patients and 20 chronic HIV-infected patients. After stimulation with HBsAg overlapping peptide, the capacity of CD8+ T cells to secrete IFN-γ was significantly improved in early HIV-infected patients (see **Figure 3A**). Furthermore, the proportion of IFN-γ in CD8+ T cells in the early HIV infection group was significantly higher than that in the chronic group (*p*<0.01) (see **Figure 3B**). The correlation analysis showed that there was no correlation between the proportion of IFN‐γ in CD8+ T cells and the CD4+ T cell counts in co-infected patients (*r*=0.070, *p*=0.837).

## 4 Discussion

The influence of HIV infection combined with HBV has been reported in many studies, and some studies have suggested that it might be due to susceptibility to HBV at different HIV infection stages (14). In this study, we explored the clinical and immunological characteristics of HIV patients from the MSM cohort coinfected with HBV in a long-term observation. From our retrospective cohort of early and chronic HIV infection studies, we found that the HBV coinfection rate increased significantly during chronic HIV infection compared with the early stage in China. HBV-specific antigens in two of four early HIV infection patients became positive as the disease developed to chronic HIV infection. From the data shown in this study, we found that the rate of HBV infection increased with the development of HIV infection. On the one hand, the lower CD4+ T cell counts and the severer dysfunction make chronic HIV-infected individuals more susceptible to HBV infection. On the other hand, the spontaneous clearance of HBV in chronic HIV-infected individuals might lead the chronic HBV infection to more likely develop due to the CD8+ T cell exhaustion spontaneous clearance ability and the reduced ability of CD8+T cells to secrete IFN-γ.

Most HBV infections are acquired during the perinatal period or due to high exposures in early childhood. In this study, we observed a clear increase in the HBV infection rate with disease development and immune system damage in adult HIV patients. The rate was significantly higher in the HIV group than in the general population (15). In China, it was reported that only 5%~10% of people above 5 years old could develop chronic HBV infection (16). The reason might be that HIV infection destroys host immune CD4+ T cells, which are resistant to HBV (17). HIV infection can cause CD4+ T cell reduction and dysfunction, making the infected human body vulnerable to HBV (17), subsequently damaging HBV clearance and ultimately aggravating the progression of liver disease (18, 19). cccDNA has been shown to persist even in the liver of patients with successful cellular and humoral control of the infection (20), which suggests that HBV may come from cccDNA in the liver tissue. Other studies have shown the reactivation of HBV after receiving anticancer therapy or rituximab-containing treatment (11, 21). Similar results in our study suggest that HIV patients are more prone to coinfection with HBV. Once infected, the spontaneous clearance ability is low, and chronic HBV infection easily develops. Due to the limitation of the number of cases, this conclusion should be tested and verified in a large-scale prospective study in the future.

Serological changes were detected in the two HIV patients infected with HBV in this study, both of whom were newly infected in the early stage of HIV. The serum of one patient was anti-HBs-positive in the early stage but confirmed as HBV-positive when HIV developed to the chronic stage. The other patient tested anti-HBc-positive only in the early stage of HIV but was HBsAg-positive in the chronic stage, which could be explained by the previous HBV infection history. Many studies have reported that spontaneous seroreversion (HBsAb negative and HBsAg conversion) often occurs in HIV-infected individuals, especially in patients whose CD4+ T cell count is below 200 cells/mm^3^ (22–24). Therefore, it suggests that it is particularly important to monitor HBsAb titre changes in HBsAb-positive patients. Vaccination may prevent vulnerable individuals from experiencing HBV reinfection. If liver function is unexplained abnormal, and the reoccurrence of HBV infection cannot be ruled out, clinicians should evaluate the stage of HBV coinfection among HIV patients. Occult Hepatitis B virus infection (OBI) is not common in the HIV-infected population, and the presence of HBV DNA replication in blood and/or liver can be detected, but serum HBsAg is negative (14). The prevalence of OBI in HIV-infected patients varies from 2% to 10% by region, since the ALT and HBV DNA levels usually increase slightly; thus, clinical OBI cannot be confirmed to be very significant. However, some studies have shown that OBI can accelerate the progression of liver disease and have suggested that monitoring HBV markers regularly and vaccination for these populations might be of great importance (25). Therefore, it is worth further exploring whether HBV positivity in these two immunocompromised HIV patients was due to the re-replication of the original HBV infection or a new infection.

Given that HIV/HBV coinfection results in higher morbidity and mortality of liver disease compared to single HBV infection, greater attention should be given to controlling HBV infection among HIV patients. As immune restoration by HAART treatment can improve HBV modulation, a preventive regimen should be taken into consideration for immune-compromised patients with HIV infection. Currently, sexual transmission among the MSM group has become the main method of HIV spread worldwide (26); the HBsAb-positive rate was less than 70% according to an investigation of the MSM group in China (27), and our study further demonstrated the importance of HBV vaccination and HBsAb detection in the MSM population.

At the early stage of infection, the response of CD8+ T cells specific for HBV plays a key role in viral clearance, but this type of response becomes weakened due to CD8+ T cell exhaustion after the disease develops into the chronic stage (28, 29). IFN-γ and TNF-α secreted by CD8+ T cells possess antiviral properties (12). Based on our observation from the early and chronic HIV patients in this study, we found that, with HIV disease progression, the number and function of CD4+ T cells decreased, and the ability of CD8+T cells to secrete IFN-γ also declined gradually. Altogether, the killing function of these cells is seriously affected. The chance of HBV reinfection is highly increased for HIV patients coinfected by HBV who do not accept ART; with the development of the disease, the HBsAb level decreases precipitously, along with the gradual impairment of immune function.

There are some limitations of this study. First, due to the limitations of our facility, we only detected the ability of CD8+ T cells to secrete IFN-γ instead of testing the killing function of HBV-infected cells directly. Second, the cohort size was relatively small, which limited our capacity to draw a robust conclusion. Third, the markers of cytotoxicity on CD8+ T cells should have been tested. However, as a pilot study, we did not detect other surrogate markers. The results of this study supported us to further explore the relationship between the cytotoxicity of CD8+ T cell after ex vivo stimulation by HBV antigens in follow-up studies. Fourth, it is a pity that the information of HBV vaccine in the two retrospective cohorts was absent. We failed to evaluate the proportion and the efficacy of HBV vaccine in the present study. The results of this study supported us to further explore the benefits in the early HIV-infected patients and chronically HIV-infected patients. In the future, we will explore the mechanism of chronic HBV infection in HIV patients if more appropriate patients become available.

In summary, the coinfection rate of HBV in HIV patients increases with HIV disease progression, which might be related to the decreased IFN-g-producing HBV-specific CD8+ T cell numbers. The closely monitored HBV serum markers from the early stage of HIV infection are warranted.

## Data Availability Statement

The raw data supporting the conclusions of this article will be made available by the authors, without undue reservation.

## Ethics Statement

The studies involving human participants were reviewed and approved by The Ethics Committee of Beijing Youan Hospital (YAH200605010). The patients/participants provided their written informed consent to participate in this study.

## Author Contributions

ZZ and YQ conceptualized the idea and drafted the manuscript. QL, WX, and MZ performed the experiments. QL, TZ, and WW conducted data analysis. TJ, HW, and YT supervised the manuscript writing. All authors read and approved the final manuscript.

## Funding

This work was supported by the National Science and Technology Major Project of the Ministry of Science and Technology of China (2018ZX10302104-002) and the Innovation Groups of the National Natural Science Foundation of China (81721002), and the National Mega-Projects of Science Research for the 13th Five-Year Plan of China (2018ZX10301407005-001 and 2018ZX10302103-001-003 to TJ).

## Conflict of Interest

The authors declare that the research was conducted in the absence of any commercial or financial relationships that could be construed as a potential conflict of interest.

## Publisher’s Note

All claims expressed in this article are solely those of the authors and do not necessarily represent those of their affiliated organizations, or those of the publisher, the editors and the reviewers. Any product that may be evaluated in this article, or claim that may be made by its manufacturer, is not guaranteed or endorsed by the publisher.

## References

[1] Jiao Y Li N Chen X Zhang T Li H Li W. Acute HIV Infection is Beneficial for Controlling Chronic Hepatitis B. Clin Infect Dis (2015) 60(1):128–34. doi: 10.1093/cid/ciu710 25205770

[2] Kim AY. Hepatitis B Virus and HIV Coinfection: Fibrosis, Fat, and Future Directions. Am J Gastroenterol (2019) 114(5):710–2. doi: 10.14309/ajg.0000000000000231 30985299

[3] Thornton AC Jose S Bhagani S Chadwick D Dunn D Gilson R. Hepatitis B, Hepatitis C, and Mortality Among HIV-Positive Individuals. AIDS (2017) 31(18):2525–32. doi: 10.1097/QAD.0000000000001646 PMC5690308 28926400

[4] Konopnicki D Mocroft A de Wit S Antunes F Ledergerber B Katlama C. Hepatitis B and HIV: Prevalence, AIDS Progression, Response to Highly Active Antiretroviral Therapy and Increased Mortality in the EuroSIDA Cohort. AIDS (2005) 19(6):593–601. doi: 10.1097/01.aids.0000163936.99401.fe 15802978

[5] Nikolopoulos GK Paraskevis D Hatzitheodorou E Moschidis Z Sypsa V Zavitsanos X. Impact of Hepatitis B Virus Infection on the Progression of AIDS and Mortality in HIV-Infected Individuals: A Cohort Study and Meta-Analysis. Clin Infect Dis (2009) 48(12):1763–71. doi: 10.1086/599110 19435436

[6] Data Collection on Adverse Events of Anti HIV drugs (D:A:D) Study Group Smith C Sabin CA Lundgren JD Thiebaut R Weber R. Factors Associated With Specific Causes of Death Amongst HIV-Positive Individuals in the D:A:D Study. AIDS (2010) 24(10):1537–48. doi: 10.1097/QAD.0b013e32833a0918 20453631

[7] Sarmati L Malagnino V. HBV Infection in HIV-Driven Immune Suppression. Viruses (2019) 11(11):1077. doi: 10.3390/v11111077 PMC6893694 31752284

[8] Fiebig EW Wright DJ Rawal BD Garrett PE Schumacher RT Peddada L. Dynamics of HIV Viremia and Antibody Seroconversion in Plasma Donors: Implications for Diagnosis and Staging of Primary HIV Infection. AIDS (2003) 17(13):1871–9. doi: 10.1097/00002030-200309050-00005 12960819

[9] Huang X Chen H Li W Li H Jin X Perelson AS. Precise Determination of Time to Reach Viral Load Set Point After Acute HIV-1 Infection. J Acquir Immune Defic Syndr (2012) 61(4):448–54. doi: 10.1097/QAI.0b013e31827146e0 PMC3705732 23143525

[10] Wang FS Fan JG Zhang Z Gao B Wang HY. The Global Burden of Liver Disease: The Major Impact of China. Hepatology (2014) 60(6):2099–108. doi: 10.1002/hep.27406 PMC4867229 25164003

[11] Ujiie I Ujiie H Yoshimoto N Iwata H Shimizu H. Prevalence of Infectious Diseases in Patients With Autoimmune Blistering Diseases. J Dermatol (2020) 47(4):378–84. doi: 10.1111/1346-8138.15244 32043652

[12] Guidotti LG Ishikawa T Hobbs MV Matzke B Schreiber R Chisari FV. Intracellular Inactivation of the Hepatitis B Virus by Cytotoxic T Lymphocytes. Immunity (1996) 4(1):25–36. doi: 10.1016/S1074-7613(00)80295-2 8574849

[13] Terrault NA Lok ASF McMahon BJ Chang KM Hwang JP Jonas MM. Update on Prevention, Diagnosis, and Treatment of Chronic Hepatitis B: AASLD 2018 Hepatitis B Guidance. Hepatology (2018) 67(4):1560–99. doi: 10.1002/hep.29800 PMC5975958 29405329

[14] Dharel N Sterling RK. Hepatitis B Virus-HIV Coinfection: Forgotten But Not Gone. Gastroenterol Hepatol (N Y) (2014) 10(12):780–8. PMC4980812 27524946

[15] Platt L French CE McGowan CR Sabin K Gower E Trickey A. Prevalence and Burden of HBV Co-Infection Among People Living With HIV: A Global Systematic Review and Meta-Analysis. J Viral Hepat (2020) 27(3):294–315. doi: 10.1111/jvh.13217 31603999 PMC7383613

[16] Xie J Han Y Qiu Z Li Y Li Y Song X. Prevalence of Hepatitis B and C Viruses in HIV-Positive Patients in China: A Cross-Sectional Study. J Int AIDS Soc (2016) 19(1):20659. doi: 10.7448/IAS.19.1.20659 26979535 PMC4793284

[17] Hadler SC Judson FN O'Malley PM Altman NL Penley K Buchbinder S. Outcome of Hepatitis B Virus Infection in Homosexual Men and its Relation to Prior Human Immunodeficiency Virus Infection. J Infect Dis (1991) 163(3):454–9. doi: 10.1093/infdis/163.3.454 1825315

[18] Parvez MK. HBV and HIV Co-Infection: Impact on Liver Pathobiology and Therapeutic Approaches. World J Hepatol (2015) 7(1):121–6. doi: 10.4254/wjh.v7.i1.121 PMC4295189 25625003

[19] Lewin SR Ribeiro RM Avihingsanon A Bowden S Matthews G Marks P. Viral Dynamics of Hepatitis B Virus DNA in Human Immunodeficiency Virus-1-Hepatitis B Virus Coinfected Individuals: Similar Effectiveness of Lamivudine, Tenofovir, or Combination Therapy. Hepatology (2009) 49(4):1113–21. doi: 10.1002/hep.22754 PMC2720274 19115219

[20] Jiao Y Zhang T Wang R Zhang H Huang X Yin J. Plasma IP-10 is Associated With Rapid Disease Progression in Early HIV-1 Infection. Viral Immunol (2012) 25(4):333–7. doi: 10.1089/vim.2012.0011 22788418

[21] Werle-Lapostolle B Bowden S Locarnini S Wursthorn K Petersen J Lau G. Persistence of cccDNA During the Natural History of Chronic Hepatitis B and Decline During Adefovir Dipivoxil Therapy. Gastroenterology (2004) 126(7):1750–8. doi: 10.1053/j.gastro.2004.03.018 15188170

[22] Biggar RJ Goedert JJ Hoofnagle J. Accelerated Loss of Antibody to Hepatitis B Surface Antigen Among Immunodeficient Homosexual Men Infected With HIV. N Engl J Med (1987) 316(10):630–1. doi: 10.1056/NEJM198703053161015 3807959

[23] Lazizi Y Grangeot-Keros L Delfraissy JF Boue F Dubreuil P Badur S. Reappearance of Hepatitis B Virus in Immune Patients Infected With the Human Immunodeficiency Virus Type 1. J Infect Dis (1988) 158(3):666–7. doi: 10.1093/infdis/158.3.666 3411157

[24] Vento S Di Perri G Garofano T Concia E Bassetti D. Reactivation of Hepatitis B in AIDS. Lancet (1989) 2(8654):108–9. doi: 10.1016/S0140-6736(89)90352-8 2567855

[25] Sulkowski MS. Viral Hepatitis and HIV Coinfection. J Hepatol (2008) 48(2):353–67. doi: 10.1016/j.jhep.2007.11.009 18155314

[26] Dong MJ Peng B Liu ZF Ye QN Liu H Lu XL. The Prevalence of HIV Among MSM in China: A Large-Scale Systematic Analysis. BMC Infect Dis (2019) 19(1):1000. doi: 10.1186/s12879-019-4559-1 31775654 PMC6880607

[27] Huang YC Hsieh SM Sheng WH Huang YS Lin KY Chen GJ. Serological Responses to Revaccination Against HBV in HIV-Positive Patients Born in the Era of Nationwide Neonatal HBV Vaccination. Liver Int (2018) 38(11):1920–9. doi: 10.1111/liv.13721 29446249

[28] Fisicaro P Barili V Montanini B Acerbi G Ferracin M Guerrieri F. Targeting Mitochondrial Dysfunction can Restore Antiviral Activity of Exhausted HBV-Specific CD8 T Cells in Chronic Hepatitis B. Nat Med (2017) 23(3):327–36. doi: 10.1038/nm.4275 28165481

[29] Wang Q Pan W Liu Y Luo J Zhu D Lu Y. Hepatitis B Virus-Specific CD8+ T Cells Maintain Functional Exhaustion After Antigen Reexposure in an Acute Activation Immune Environment. Front Immunol (2018) 9:219. doi: 10.3389/fimmu.2018.00219 29483916 PMC5816053

